# FEASIBILITY OF A VIRTUAL CARE FARM FOR OLDER ADULTS WITH COGNITIVE AND PHYSICAL LIMITATIONS

**DOI:** 10.1093/geroni/igae098.2046

**Published:** 2024-12-31

**Authors:** Keith Anderson, Rebecca Mauldin, Megan Westmore, Anna Tulloh

**Affiliations:** University of Mississippi, Oxford, Mississippi, United States; The University of Texas Arlington, Arlington, Texas, United States; The University of Texas Arlington, Arlington, Texas, United States; The University of Texas Arlington, Arlington, Texas, United States

## Abstract

Nature-based activities provide an array of physical, emotional, social, and spiritual benefits for people of all ages. Older adults with cognitive and physical limitations, however, are often excluded from these activities due to environmental, functional, and financial barriers. In this study, the researchers evaluated the feasibility of delivering a virtual (online), nature-based, group activity to older adults with physical and cognitive limitations in assisted living. Days at Dunrovin (D@D) is a “virtual care farm” featuring live web-cameras, recorded content, and online discussion boards that focus on life on a rural Montana ranch. Qualitative data from 18 interviews with participants, staff, and interventionists and 40 days of field notes were analyzed for themes related to feasibility. Themes were organized using an established framework of feasibility domains and outcomes. Results indicated that D@D was well-received by participants (Demand/Acceptability). Results also illuminated the process by which the researchers delivered and subsequently modified the activity (Implementation/Adaptation) in response to challenges presented by the assisted living environment, including the cognitive functioning of participants, the physical structure of the facility, and staffing issues (Practicality/Integration). A range of benefits (Effectiveness) were associated with D@D, such as gains in emotional well-being, increased socialization, enhanced engagement with nature, and stimulation of reminiscence. These findings suggest that it is feasible to deliver virtual, nature-based group activities to older adults with cognitive and physical limitations in long-term care. In doing so, we may be able to provide expanded access to the wonders of nature to this all too often excluded population.

